# Establishing a culturally specific nursing home for Finnish‐speaking older persons in Sweden: A case study

**DOI:** 10.1002/nop2.129

**Published:** 2018-02-23

**Authors:** Emina Hadziabdic, Katarina Hjelm

**Affiliations:** ^1^ Faculty of Health and Life Sciences Department of Health and Caring Sciences Linnaeus University Växjö Sweden; ^2^ Department of Social and Welfare Studies Linkoping University Linköping Sweden; ^3^ Department of Public Health and Caring Sciences Uppsala University Uppsala Sweden

**Keywords:** case study, cultural diversity, culturally specific nursing home, older migrants, transcultural nursing

## Abstract

**Aim:**

The study aims to describe the establishment of a culturally specific nursing home for Finnish‐speaking older persons in Sweden.

**Design:**

A descriptive qualitative study.

**Methods:**

A descriptive case study based on a review of 14 public documents and individual interviews with two experts in the area, analysed with qualitative content analysis.

**Results:**

This study found that shared language, preservation of customs and habits and collaboration between the representatives of the municipality, Finnish‐speaking migrant associations and staff at the nursing home influenced the development of the culturally specific nursing home for older Finnish‐speaking people intended to avoid loneliness, isolation and misunderstandings among older Finnish‐speaking. Collaboration between healthcare service for older persons and minority people resulted in an optimal culturally specific nursing home, simultaneously encountering the majority culture. Nursing and healthcare services need to be aware of positive effects of collaboration with stakeholders to achieve optimal culturally specific nursing homes.

## 1 INTRODUCTION

Worldwide there are growing multicultural and multilingual societies as a result of global migration. There are an estimated 232 million international migrants and this number is expected to increase (Lee, 2015). Today 16% of the Swedish population are born abroad and migrants in Sweden are a heterogeneous group of persons due to the migration pattern that has consisted of different waves: 1) starting in the postwar period and in the 1960–1970s with voluntary labour migrants mainly from the Scandinavian countries, especially Finland; 2) during the 1990s with refugee immigration, mainly from the former Yugoslavia and the Middle East and 3) up to today's wave of refugees from Afghanistan, North Africa and Syria (Statistics of Sweden, 2016). In Sweden, there are approximately 1.75 million older people (over 65 years) of whom around 12% were born abroad. However, the largest group of migrants in Sweden is Finns (Statistics of Sweden, 2016) and the majority of Finns have never learnt Swedish due to their labour migration history (Heikkila & Ekman, 2000). The increasing age of the older migrant population who do not speak the language of the host country makes it necessary for older healthcare services to meet the healthcare needs concerning the language and cultural barriers (National Board of Health and Welfare, 2007; The Department of Care and Care Analysis, 2015) because healthcare settings for older people are usually planned and organized to suit the needs of the majority population (National Board of Health and Welfare, 2015). Culturally specific nursing homes for minority seniors focus mostly on traditional care, where healthcare staff speak the residential language, the meals are culturally appropriate, residents' religion is provided for and there is an understanding of specific holidays and customs (Olson, 2001). Thus, this study focuses on the establishment of a culturally specific nursing home for Finnish‐speaking older persons in a specific migrant area in Sweden.

## 2 BACKGROUND

A previous study (Hovde, Hallberg, & Edberg, 2008a) found that older immigrants have poorer health and that this is mainly related to socio‐economic factors. Yet, they often receive less health care than native Swedes (Hovde et al., 2008a) because they receive more informal help from their families, they have more behaviour perceived to be difficult to handle (Hovde, Hallberg, & Edberg, 2008b) and they need a more culturally adopted appropriate healthcare setting (Chan & Kayser‐Jones, 2005; Heikkila & Ekman, 2003).

Previously, research focusing on culturally specific nursing homes for migrant seniors has not been undertaken with the exception of two studies (Andrews, 2012; Heikkila, Sarvimaki, & Ekman, 2007). One of the previous studies (Heikkila et al., 2007) focused on how cultural congruency was used in the care of older Finnish immigrants in a nursing home in Sweden and showed that mother language, a shared ethnic background with the staff and shared customs support a caring relationship, which in turn increases the older people's well‐being. The other study, conducted in Australia, investigated what made Anglo‐Indian residential care homes distinctive as a reason for moving into a home (Andrews, 2012). Anglo‐Indian residential care was viewed as supporting traditional Anglo‐Indian customs through the way the homes were run, which allowed the residents to live the daily life of an Anglo‐Indian in their old age.

Research concerning culturally specific nursing homes for senior migrants is scarce and highly needed in an increasingly globalized world, if we are to develop culturally specific homes in the future to promote culturally appropriate health care for older migrants. Because the number of ageing foreign‐born people is gradually increasing and language and cultural differences make high demands on the adaptation of older health care, it is important to investigate the way a culturally specific nursing home for Finnish‐speaking older people was established in Sweden to obtain a better understanding of the subject. In this study, the focus was on a municipality in Sweden, where there is a Finnish administrative area where Finnish‐speaking people have a special position with rights to use their native Finnish language in contact with authorities (Swedish Parliament, 2009:724). This law, along with the Social Services Act (Swedish Parliament, 2001:453) and the Patients Act (Swedish Parliament, 2014:821), emphasizes that healthcare services for older persons should be adapted to the needs of older people. The authorities will therefore ensure that there are Finnish‐speaking healthcare staff in the Finnish administrative areas. To achieve the statutory goals and intentions in the municipality's diversity plan (Municipality's health and social care board, 2011), the municipality started a so‐called “elderly nursing home” for Finnish‐speaking older persons in December 2013. The purpose of the culturally specific nursing home for Finnish‐speaking older persons was that needs for language, food and cultural activities together with Swedish culture must be satisfied. However, there is no knowledge about the expectations and wishes that existed among decision makers, Finnish‐speaking older persons and their relatives and healthcare professionals during the establishment of a culturally specific nursing home. This is important to document, as well as the process from idea to implementation, to obtain practical advice that can facilitate the development of future older nursing homes adapted to older migrants of other origins than Finnish‐speaking.

## 3 AIM

The aim of the study was to describe establishment of a culturally specific nursing home for Finnish‐speaking older persons in Sweden.

## 4 DESIGN

This study is a descriptive qualitative study based on a review of public documents and individual interviews from a case study of the establishment of a culturally specific nursing home in a migrant‐dense area, to develop a deeper understanding of a real‐life case and investigate what can be learnt about the phenomenon (Patton, 2015; Stake, 1995). Public documents concerning the development of the home and individual interviews with key people, who are experts as they have specific knowledge of the development of the home and the organizations, were used because they are rich and valuable sources of information to ascertain how the programme proceeded over time (Stake, 1995; Yin, 2014).

## 5 METHOD

### 5.1 Sample and setting

A municipality that belonged to a Finnish administrative area in Sweden was studied. The studied municipality served approximately 24 607 older people (aged over 65), of whom 769 persons were older Finnish‐born persons (Stastitics of Sweden, 2017). The Swedish laws (Swedish Parliament, 1982:763, 2009:724, Patientlag (Patient Act), 2001:821) emphasize that healthcare services should be adapted to older people's specific needs. Thus, authorities in the Finnish‐speaking administrative area have to ensure that healthcare staff with skills in the Finnish language are to be found in the municipality, particularly in the care of older persons. The culturally specific nursing home is regulated by Swedish laws and rules (Swedish Parliament, 1982:763, 2001:453, Patientlag (Patient Act), 2001:821) for running nursing homes in Sweden and the home has requirements from the Finnish administrative area to fulfil the needs of older people with Finnish background to have their language, food and culture together with the Swedish culture (Municipality's health and social care board, 2011).

### 5.2 Procedure

The project coordinator and the project manager for the Finnish administrative area in the municipality contacted the researchers (EH and KH) and expressed their wish to investigate documents concerning how a culturally specific nursing home for people with a Finnish‐speaking background was established. Copies of the public reports such as minutes of meetings, decisions from the meetings, reports and applications were then handed over to the first author (EH) and a time was set for interviews with persons who had been involved in the development of the culturally specific nursing home for Finnish‐speaking older.

The study included 14 public documents written by the business controller, municipal secretary, project coordinator and project manager for the Finnish administrative area of community health care and a student during the years 2011–2015 and two persons (females) who had experience of planning, starting and organizing the culturally specific home for Finnish‐speaking persons, a procedure that had lasted about 4 years.

### 5.3 Data collection

Data were collected during the years 2015 and 2016 by reviewing public documents concerning the development of a culturally specific nursing home and semi‐structured individual interviews with key people having expert knowledge of the development of the home and the organization.

The public documents described the establishment of the Finnish‐speaking nursing home in a Swedish municipality and they included minutes of meetings of the city council, applications to the government office, decisions by the government office and reports to the city council. The business controller, municipal secretary, project coordinator and project manager for the Finnish administrative area of community health care and a student wrote public documents and they included a statement of the year, month and day.

Expert interviews were held with two persons with particular knowledge of the development process, following an interview guide based on public documents. The interview guide focused on experiences of planning, starting and organizing the culturally specific home for Finnish‐speaking persons. The interviews were held by a researcher experienced in migrants' health and healthcare issues (first author) by telephone and lasted approximately 60 min. All interviews were audiotaped and transcribed verbatim by a professional secretary and then analysed by the first author.

### 5.4 Data analysis

Qualitative content analysis was used to categorize essential regularities and meanings in the data related to the aim of the research (Patton, 2015). First, the texts were read through several times to obtain a sense of the whole. Secondly, the texts were sorted and then compared for differences and similarities and finally grouped into three areas: 1) the start; 2) economy; and 3) organization, activities and service.

### 5.5 Rigour and Trustworthiness

The following steps were taken to make the study more rigorous (Patton, 2015):

- to ensure credibility: constant reading and reflection were used throughout the analytical process, the categories were defined and named as closely as possible to the text, data and investigator triangulation using different data collection methods and both authors experienced in qualitative studies and migrants health and healthcare issues analysing the material and comparing the results to confirm areas of relevance
- to ensure dependability: the methodological process was described as clearly as possible (Patton, 2015).

### 5.6 Ethics

According to the Swedish law concerning the regulation of ethical research involving humans (Swedish Parliament, 2003:460), approval by an official research committee was not required as the study posed no physical or mental danger and did not disclose participants' data concerning personal matters or health. The study was implemented according to the ethical principles stated in the Declaration of Helsinki (WMA (World Medical Association), 2013), eg, risk/benefit assessment, informed consent and participants' authorization, confidentiality and respect for human dignity and the Swedish law in terms of the regulation of ethics in research involving people (Swedish Parliament, 2003:460).

## 6 RESULTS

Three areas emerged from the data: 1) push factors for the establishment of the culture‐specific nursing home; 2) the culturally specific nursing homes organization, activities and service and 3) outcomes of the services.

### 6.1 Push factors for the establishment of the culture‐specific nursing home

The Swedish‐Finnish‐speaking community in the Swedish church and Finnish‐speaking persons associations in the municipality expressed a desire through petitions to the municipality leaders to apply to become an administrative area for Finnish‐speaking persons. Furthermore, the municipal executive and council decided to send the request for the municipality to become an administrative area for Finnish‐speaking persons to the Swedish government. The Swedish government then decided that the municipality should be part of the Finnish‐speaking people administrative area starting from January 2012. To operate activities in the Finnish‐speaking administrative area, the municipality received government grants each year.

#### 6.1.1 Economy in the Finnish administrative area in the municipality

The municipal grants from the state in 2012–2015 amounted to SEK 1.98 million. The year 2012 showed a surplus of SEK 452 000 because the community healthcare and education offices had not yet started with the activities. However, the years 2013–2014 showed a deficit (30 036, 90 SEK), partly because the community health care had a project manager hired by the Finnish‐speaking administrative area funds.

#### 6.1.2 Organization

It was an initiative of the healthcare department in the municipality to start the culturally specific nursing home for Finnish‐speaking persons. They started first with a survey of all Finnish‐born persons aged over 70 to find out their desires and needs regarding older healthcare services in 2013 and in the future. The survey was sent to 610 persons and 362 people responded, of whom 78% said that they wanted to stay at a nursing home completely or partly run in Finnish with well‐trained Finnish‐speaking staff because of the risk that older people remember only their mother tongue in association with dementia. Furthermore, the respondents' desire was that the nursing home should be located in an older community healthcare department or residential house close to nature, with premises for fitness and sauna and close to transport facilities and supermarkets.

The head of the healthcare department in the municipality, the project coordinator and the head of the future nursing home and representatives of Finnish‐speaking associations held the planning and organizing of the Finnish‐speaking person's home. The collaboration between the above‐mentioned partners and the needs/desires of the older to be placed in the culturally specific nursing home, but also the needs of the older to be placed in nursing homes in community healthcare were factors that had an impact on the development of the culturally specific home for Finnish‐speaking people. Only a limited number of older Finnish‐speaking persons reported their interest in being a resident in the Finnish culturally specific nursing home, so it was decided to organize the home with a mixture of Swedes and Finns.

The next step in the organizing was that the municipality sent an inquiry concerning which nursing home that was able to develop in to a cultural congruent nursing home for Finnish‐speaking people. The inquiry was sent to both public and privately run nursing homes in the municipality. Only one privately run nursing home responded to the inquiry and the municipality helped with funding received from the Swedish government to develop it into a culturally specific nursing home for Finnish‐speaking persons.

The culturally specific nursing home started in 2013 with the requirements to fulfil the needs of older people with a Finnish‐speaking background as regards language, food and culture, together with the Swedish culture. Furthermore, the department was organized as a mixture of Swedish and Finnish‐speaking residents because there were not enough Finnish‐speaking applicants for culturally specific nursing home. At the start, there were three care recipients living in the nursing home. At present, there are six care recipients living in the culturally specific nursing home.

### 6.2 The culturally specific nursing home's organization, activities and service

The culturally specific nursing home is organized with Finnish activities presented on Wednesdays from 12:00–12:30. The meeting place is furnished in Finnish style, with the help of donations of Finnish‐speaking individuals and the Finnish‐speaking Association. Finnish‐speaking pensioners and Finnish associations are engaged in activities with singing, baking and cooking to mark holidays, such as eg, a Christmas party on December 4 and celebrating the Finnish Independence Day on December 6. In 2013 and 2014 the nursing home reported difficulties to start the meeting place and implementation of various Finnish activities. It was found in public documentation that two of the residents felt that there were not enough Finnish activities available at the nursing home and they wished for more frequent Finnish activities. To fulfil the language requirement, the culturally specific nursing home has Finnish‐speaking healthcare staff such as a registered nurse and two assistant nurses.

### 6.3 Outcomes of the services

The positive experiences of the organization, activities and service related to the culturally specific nursing home were that the healthcare department in the municipality supported the establishment process of a culturally specific nursing home. Other positive experiences were that collaboration with healthcare staff at the nursing home was good; there is constant development to improve diet and cultural activities and greater possibility for the Finnish‐speaking older to speak their mother tongue.

The dissatisfaction was found regarding the availability of Finnish‐speaking older care from a member of the Finnish‐speaking association. It was described that there was a lack of persons speaking Finnish at the culturally specific nursing home. However, the culturally specific nursing home was felt to be a good nursing home, but making room for all Finnish speakers who want to live there is a problem. The desire was that all older Finnish‐speaking persons should stay in the same culturally specific nursing home. Other negative experiences of the project start were that the project leaders experienced a slow start because the administrators and politicians in the municipality lacked knowledge of minority administrative areas, as a result of which the project leaders did not get the necessary response for their work of developing the a specific culturally nursing home. Furthermore, Finnish‐speaking associations experienced that the start of the home took too long to develop and they questioned the municipality's competence in Sweden‐Finnish history, working methods and working hours. It was also found that it was difficult to recruit Finnish‐speaking staff for the profiled nursing home.

## 7 DISCUSSION

In this study, not previously described, factors such as shared language, preservation of customs and habits, collaboration between representatives of the municipality, Finnish‐speaking associations and the nursing home influenced the development of the nursing home for older Finnish‐speaking people. The organization of the culturally specific nursing home emphasizes the need to provide culturally congruent care based on the group's cultural beliefs, practices and values (Leininger & McFarland, 2006). Other important aspects of care in this study were residents' opportunities to share a common language with other older and to express their needs to the healthcare staff. These findings mirror those of previous studies (Andrews, 2012; Heikkila & Ekman, 2003; Heikkila et al., 2007) about the opportunity to speak the mother tongue in a familiar socio‐cultural environment to avoid feelings of loneliness, isolation and misunderstanding. In addition to language, to be understood and to understand, cultural content is an important part of the identity and therefore also important for the well‐being of older persons (Emami, Torres, Lipson, & Ekman, 2000; National Board of Health and Welfare, 2015).

Patient safety as well as quality of health care emphasize that healthcare service for older persons should be adapted to older people's specific needs and delivered on equal terms and with patient participation in the health care (Swedish Parliament, 1982:763, 2001:453, 2001:821; World Health Organization. & United Nations. Office of the High Commissioner for Human Rights, 2008). Despite these recommendations, older born abroad often live in ordinary nursing homes that is not adapted to the possibilities to be equally involved in the health care because of possibilities to communicate in the term of language (Chan & Kayser‐Jones, 2005; Hadziabdic, Lundin, & Hjelm, 2015; National Board of Health and Welfare, 2015; Plejert, Jansson, & Yazdanpanah, 2014) lack of adjustment of cultural beliefs and customs (Chan & Kayser‐Jones, 2005; National Board of Health and Welfare, 2015), lack of requests for older health care in the minority language, municipalities' lack of knowledge about the law on national minorities and lack of information translated into minority languages targeted to the older and their families (National Board of Health and Welfare, 2015). Healthcare services for older persons needs to be adapted to the growing diversity of older people, otherwise it will run the risk that foreign‐born older avoid formal care and instead rely on informal healthcare from the family, resulting in a heavier health care burden for the families (Hovde et al., 2008a, 2008b; The Department of Care and Care Analysis, 2015). Previous studies (Andrews, 2012; Heikkila & Ekman, 2003; National Board of Health and Welfare, 2015) have found that older members of minority communities who live in institutional older health care, where they can receive culturally appropriate care in familiar socio‐cultural circumstances increases the well‐being of older people. The new findings highlight the need for culturally specific nursing homes for older members of minority communities, where an option could be this solution of the culturally specific nursing home, including healthcare staff speaking the minority language, organized as a mixture of older members of both the majority and the minority communities living in the same nursing home, which allows all residents have possibilities to share language and preserve customs and habits. The possibility of good communication and adjustment of cultural beliefs and customs requires older minority residents to be involved in health care and able to participate in decision‐making concerning health care to deliver individualized and high‐quality holistic health care (Swedish Parliament, 1982:763, 2001:821).

### 7.1 Strengths and limitations of the study

The strategy for the study was based on a case study. The advantage was that this provided insight and knowledge of the phenomenon under study from a real‐life case (Stake, 1995). The findings are thus contextual and cannot be generalized as they involve an understanding of the particular case, but as two different data sources gave a similar picture of the information, the findings can be transferred to other contexts with similar characteristics (Patton, 2015; Stake, 1995).

The other strategy for the study was to use two different data sources, for example, review of public documents and individual interviews, to report a broader range of historical, attitudinal and behavioural concerns (Yin, 2014). The advantage of using public documents includes efficiency and cost‐effectiveness to obtain empirical data as a part of a research process that is unobtrusive and non‐reactive (Bowen, 2009; Yin, 2014). Potential problems with the chosen data collection methods could be that: 1) documents were produced for other reasons than research and 2) selected documents were likely to be associated with policies and procedures and with the plan of the organization's leaders. However, the documents were useful in this study for giving an opportunity to investigate the way a culturally specific nursing home for older minority people was established and to cover a frame period of 4 years with several stakeholders involved (Bowen, 2009; Yin, 2014). In qualitative studies, sample size depends on the purpose of the inquiry, what will be useful and what will have credibility. Thus, the rationale to interview two people with particular knowledge of the development process was to get additional in depth‐and rich information about the particular case and to strengthen triangulation by mixing data collection methods (Patton, 2015).

## 8 CONCLUSION AND IMPLICATIONS

This case study showed that factors such as shared language, preservation of customs and habits and collaboration between the representatives of the municipality, Finnish migrant associations and staff at the nursing home influenced the development of the culturally specific nursing home for older Finnish‐ speaking people. For minority seniors, this may lead to avoidance of loneliness, isolation and misunderstandings, which in turn increase the well‐being of older people. Nurses and healthcare service can improve well‐being for minority seniors by working collaboratively with minority people to achieve optimal culturally specific nursing homes as regards language, food and culture while also encountering the majority culture.

## CONFLICT OF INTEREST

The authors declared no potential conflicts of interest with respect to the research, authorship, and/or publication of this article.

## AUTHOR CONTRIBUTIONS

EH, KH, Study design; EH, Data collection; EH, Data analysis; EH;KH, Study supervision; EH, KH, Manuscript writing; EH, KH, Critical revisions for important intellectual content.
